# Adults’ willingness to report sexual orientation and gender identity when registering for a digital health application: A cross-sectional quantitative study

**DOI:** 10.1371/journal.pone.0292739

**Published:** 2023-11-20

**Authors:** Jaclyn Marshall, Xinyu Zhang, Benjamin B. Green

**Affiliations:** Included Health, San Francisco, CA, United States of America; Johns Hopkins University Bloomberg School of Public Health, UNITED STATES

## Abstract

The collection of patient sexual orientation and gender identity information is crucial in identifying and addressing disparities in healthcare access, quality, and outcomes for sexual and gender minority individuals. While some studies have explored patients’ willingness to disclose this information in specific settings, little is known about response rates in digital health applications. In light of the growing use of digital health, including virtual care, we sought to determine whether adults would respond to optional sexual orientation and gender identity fields during registration for a digital health application offered through their employer-provided benefits. We analyzed response rates for sexual orientation and gender identity by age, race and ethnicity, and region among individuals over age 17 between September 9th and December 31, 2022. Our study, which included over 41,000 commercially-insured adults from all 50 states, found that nearly 80% were willing to report their sexual orientation and gender identity. However, we observed higher nonresponse rates among older adults and individuals living in central and southern regions, with no consistent pattern by race and ethnicity. Our findings indicate that digital health applications could be a valuable resource for collecting this data from a diverse group of adults. Nevertheless, digital health companies must ensure that they use the data responsibly, identifying quality improvement initiatives and contributing to research that can inform health policies for sexual and gender minority individuals.

## Introduction

Members of sexual and gender minority (SGM) communities are less likely to access health care, have higher prevalence of chronic diseases, and subsequently worse health outcomes compared to the general population [1,2]. To improve the health of individuals in SGM communities, it is crucial to conduct further research to better understand the disparities in access, utilization, and outcomes. This knowledge can inform quality improvement initiatives and health policies, such as coverage of services used primarily by SGM communities or implementing payment models tied to reducing disparities for this community [3]. However, the lack of data that identifies members in SGM communities remains a grave limitation and further exacerbates the dearth of literature around the impact of health crises, such as COVID, on SGM populations [4].

For over a decade, the Federal government has issued reports and policies to encourage the standardized collection of sexual orientation and gender identity (SOGI) information from patients. In 2011, the Institute of Medicine and the Joint Commission highlighted the need to collect standardized SOGI data in electronic health records to better understand this population [2,5]. Subsequently, the Office of the National Coordinator for Health Information Technology required electronic health records to enable collection of SOGI to meet Meaningful Use Stage 3 criteria in 2015, and the Health Resources and Services Administration required Federally Qualified Health Centers to collect SOGI data from adult patients in 2016 [6]. Most recently, in 2022, the National Academies published recommendations for collecting and measuring sex, gender identity, and sexual orientation through standardized language [7]. Despite these efforts, and calls from providers, researchers, and advocates, there are currently no national requirements across providers and health care organizations to collect standardized patient SOGI information [8–10].

While research has shown that patients are willing to share this information, especially in outpatient settings, it is unknown if they would be willing to report SOGI data through digital health applications [11–14]. Digital health is a broad term that encompasses the use of technology in medicine and the healthcare field, and includes products and services such as mobile health applications, virtual health care delivery via telemedicine, wearables, and general wellness applications [15]. With the digital health market projected to grow, and specifically the increased use of virtual health care delivery since the pandemic, digital health applications may provide an effective means to collect SOGI data from a broad group of patients [16,17].

The purpose of this study was to determine the likelihood of adults to provide their sexual orientation and gender identity during the enrollment process of a digital health application. It also sought to test our hypotheses that SOGI response rates are lower among members in older age groups, members who reside in the southern and central regions of the US, and are not White [11,18–20].

## Methods

This cross-sectional study utilized de-identified data from a digital health application registration and eligibility database, ensuring that the authors did not have access to any identifiable information about the members. As part of the registration process, each member agrees to our data privacy policy, which includes a provision that their de-identified data may be used for research purposes. The WIRB-Copernicus Group (WCG) IRB considered this study exempt from institutional review board approval in accordance with 45 CFR §46.102. This study follows the Strengthening the Reporting of Observational Studies in Epidemiology (STROBE) reporting guidelines (S1 File).

### Digital health application and study sample

The digital health application includes both health care navigation and virtual care services offered as an employer-provided benefit to employees and their dependents (‘members’). Specific services available to members may differ by employer. Health care navigation services are available via self-service, in-app messaging, or live telephonic support with the care coordinator team. Health care navigation services include claims advocacy, clinical guidance, triage, expert medical opinion, high-quality provider referral and appointment scheduling, benefits routing, a financial toolkit, care and case management, and specialized navigation for members who identify as Lesbian, Gay, Bisexual, Transgender, and Queer (LGBTQ+) or within the Black population. Virtual care services include urgent care, primary care, and behavioral health care delivered via video by a team of employed clinicians.

Employers who have offered services through the digital health application to their members provide details about the services provided, as well as instructions on the registration process. They also disseminate information regarding the application in a variety of ways, such as the benefits guide, email, mailers, or through employee resource groups.

Members may register for the application at any point after the employer begins to cover the benefit. As of September 9th, 2022, the application began collecting optional demographic information during the registration process. Our study sample included all members over 17 years old who registered in the application on or after September 9th through December 31, 2022. During this time period, there were approximately 313,000 members who were eligible for services and had the opportunity to register.

### Optional questions during registration

During registration, the member was required to provide their name and a unique identifier, such as their birth date or health insurance member ID, to confirm eligibility for the application. If they chose to continue, they were presented with four optional fields to ’personalize their care’: race and ethnicity, pronouns, sexual orientation, and gender identity (Table 1). The SOGI questions and response options were informed by validated instruments and expanded for greater inclusivity [10,21,22].

**Table 1:** Optional questions and response options during digital health application registration.

How do you describe yourself? Please select all that apply. • American Indian or Alaska Native • Asian • Black or African American • Native Hawaiian or Other Pacific Islander • White • Hispanic • My race is not listed [open text field] • I prefer not to answer
What are your pronouns? Please select all that apply. • he/him/his • she/her/hers • they/them/theirs • ze/hir/hirs • My pronouns are not listed [open text field] • I prefer not to answer
What’s your gender? • Cisgender man • Cisgender woman • Genderfluid • Nonbinary • Transgender man • Transgender woman • Agender • My gender is not listed [open text field] • I prefer not to answer
How do you identify? Please select all that apply. • Aromantic • Asexual • Bisexual • Gay • Heterosexual • Lesbian • Pansexual • Queer • My identity is not listed [open text field] • I prefer not to answer

### Variables

The variables included member responses to the four optional questions above and member age and geographic location from the employer eligibility files. For race and ethnicity, sexual orientation, and pronouns, members were allowed to choose multiple response options. In these cases, we categorized the response as ’multiple select’ and did not include the responses within the categories they selected. We made this analytical decision to ensure mutually exclusive groups for statistical testing. In addition, members who chose ‘my race/pronouns/gender/identity is not listed’ and then entered free text that was similar to a predefined option, were not recategorized. The responses remained coded as ‘my race/pronouns/gender/identity is not listed’. Members who chose to not answer the question (missing values) were coded as ‘null response’.

The primary objective of this study was to determine how likely adult members were to report their sexual orientation and gender identity. Therefore, we defined item nonresponse as anyone who responded ‘I prefer not to answer’ or had a ‘null response’. The nonresponse rate was calculated as the proportion of members who had a nonresponse divided by all members who chose to continue to the optional questions. Age was defined as 18 to 26, 27 to 35, 36 to 45, 46 to 55, 56 to 64, or 65+. Member geography was defined by the 9 U.S. Census regions [23].

### Analysis

We used counts and proportions to report member demographic characteristics and the responses to the four optional questions. We calculated chi-square statistics to test for correlation in item nonresponse (‘I prefer not to answer’ or ‘null response’) across race and ethnicity, gender identity, and sexual orientation questions. We also used chi-square statistics to test for differences in race and ethnicity, age, and geographic location between members who responded to individual SOGI questions and members who did not. We calculated nonresponse rates overall and separately by race and ethnicity, age, and geographic location. Finally, understanding the drivers of responding ‘I prefer not to answer’ and ‘null response’ may differ, we also conducted a sensitivity test that removed ‘I prefer not to answer’ responses from the nonresponse group and recalculated the chi-square statistic between respondents and nonrespondents to see if any differences found in the primary comparison remained. Alpha was defined as p<0.05.

## Results

### Member demographics and responses to optional fields when registering for the digital health application

Overall, 113,064 new members registered during the study period and 41,677 (36.8%) chose to continue to the optional questions. Table 2 includes the responses to the questions and the member demographic characteristics among those presented with the optional questions.

**Table 2:** Demographic characteristics of newly registered members who continued to optional questions and member responses to race and ethnicity, sexual orientation, gender identity, and pronoun questions during digital health application registration between September 9th and December 31, 2022.

	N. (%)
Newly registered members who continued to optional questions	41677 (100%)
Race and ethnicity	
American Indian or Alaska Native	124 (0.3%)
Asian	12068 (29%)
Black or African American	2515 (6%)
Hispanic	2832 (6.8%)
Native Hawaiian or Other Pacific Islander	47 (0.1%)
White	17385 (41.7%)
Multiple selected^a^	1540 (3.7%)
My race is not listed	543 (1.3%)
I prefer not to answer	2071 (5%)
Null response	2552 (6.1%)
Pronouns	
He/him/his	21451 (51.5%)
She/her/hers	16334 (39.2%)
They/them/theirs	148 (0.4%)
Ze/hir/hirs	2 (0%)
Multiple selected^b^	457 (1.1%)
My pronouns are not listed	82 (0.2%)
I prefer not to answer	104 (0.2%)
Null response	3099 (7.4%)
Gender identity	
Cisgender man	19083 (45.8%)
Cisgender woman	13747 (33%)
Genderfluid	105 (0.3%)
Non-binary	226 (0.5%)
Transgender man	65 (0.2%)
Transgender woman	125 (0.3%)
Agender	47 (0.1%)
My gender is not listed	481 (1.2%)
I prefer not to answer	2216 (5.3%)
Null response	5582 (13.4%)
Sexual orientation	
Aromantic	35 (0.1%)
Asexual	131 (0.3%)
Bisexual	912 (2.2%)
Gay	1098 (2.6%)
Heterosexual	30250 (72.6%)
Lesbian	335 (0.8%)
Pansexual	223 (0.5%)
Queer	197 (0.5%)
Multiple selected^c^	634 (1.5%)
My identity is not listed	158 (0.4%)
I prefer not to answer	3277 (7.9%)
Null response	4427 (10.6%)
Age tier at registration, y	
18–26	5144 (12.3%)
27–35	15577 (37.4%)
36–45	12589 (30.2%)
46–55	5929 (14.2%)
56–64	2149 (5.2%)
65+	289 (0.7%)
Geographic region	
East North Central	2768 (6.6%)
East South Central	925 (2.2%)
Middle Atlantic	5527 (13.3%)
Mountain	1902 (4.6%)
New England	1355 (3.3%)
Pacific	20299 (48.7%)
South Atlantic	4320 (10.4%)
West North Central	1108 (2.7%)
West South Central	3473 (8.3%)

SOURCE Authors’ analysis of member responses to optional questions during digital health application registration and member age and geographic location information available in the employer eligibility files. Members who provided more than one response for race and ethnicity, pronouns, and sexual orientation were coded as ‘Multiple select’.Members who did not select a response were coded as ‘null response’.

^a^Approximately 77% identified as White, 38% Hispanic, 26% Black or African American, 39% Asian, 23% Native Hawaiian or other Pacific Islander, and 16% American Indian or Alaska Native.

^b^Approximately 64% chose ‘she/her/hers’, and 75% chose ‘they/them/theirs’.

^c^Approximately 52% identified as bisexual, 52% as queer, 28% as heterosexual, and 20% as lesbian.

Over 67% of the members who were prompted to complete the optional fields were between the ages of 27 and 45 years old and almost half lived in the Pacific region of the United States.

Approximately 42% of members identified as White, 29% as Asian, 6% as Black or African American, 7% as Hispanic, 4% selected multiple races and ethnicities, and 1% responded ‘my race is not listed’. The race and ethnicity nonresponse rate was 11%; 5% chose ‘I prefer not to answer’ and 6% had a null response.

Fifty-one percent of members selected he/him/his pronouns, 39% chose she/her/hers, and 1.1% selected multiple responses. The nonresponse rate was 7.7%; 0.25% chose ‘I prefer not to answer’ and 7% had a null response.

Over 79% of members’ gender identity was cisgender, 1.2% of members’ gender was not listed, and 1.4% identified as transgender, agender, genderfluid or non-binary. The nonresponse rate was 18.7%; 5.3% chose ‘I prefer not to answer’ and 13.4% had a null response.

Almost 73% of members identified as heterosexual, 3.4% as lesbian or gay, 2.2% as bisexual, and 1.4% identified as either aromantic, asexual, pansexual, or queer. Over 18% did not provide their sexual orientation; 7.9% chose ‘I prefer not to answer’ and 10.6% had a null response.

We found that members who had a nonresponse to race and ethnicity were more likely to have a nonresponse to sexual orientation and gender identity (p<0.001). Similarly, members who had a nonresponse to sexual orientation were more likely to have a nonresponse to gender identity (p<0.001).

### Differences in sexual orientation and gender identity nonresponse by member demographics

Table 3 displays nonresponse rates by member demographics and compares characteristics of sexual orientation and gender identity respondents to nonrespondents. Respondents who provided information about their sexual orientation and gender identity exhibited differences from nonrespondents in terms of race and ethnicity, age, and geographic region (p<0.001). SOGI nonresponse rates were higher among older members, members residing in the central and southern U.S regions, and varied across different races and ethnicities.

**Table 3:** Characteristics of gender identity and sexual orientation respondents and nonrespondents, and nonresponse rates by characteristic between September 9th and December 31, 2022.

	Gender identity		Gender identity nonresponse rate (%)	Sexual orientation		Sexual orientation nonresponse rate (%)
	Respondents N (col %)	Nonrespondents N (col %)		Respondents N (col %)	Nonrespondents N (col %)	
All newly registered members who continued to optional questions	33879 (100%)	7798 (100%)	18.7%	33973 (100%)	7704 (100%)	18.5%
Race/ethnicity						
American Indian or Alaska Native	90 (0.3%)	34 (0.4%)	27.4%	100 (0.3%)	24 (0.3%)	19.4%
Asian	10786 (31.8%)	1282 (16.4%)	10.6%	10303 (30.3%)	1765 (22.9%)	14.6%
Black or African American	2072 (6.1%)	443 (5.7%)	17.6%	2139 (6.3%)	376 (4.9%)	15.0%
Hispanic	2378 (7%)	454 (5.8%)	16.0%	2488 (7.3%)	344 (4.5%)	12.1%
Native Hawaiian or Other Pacific Islander	39 (0.1%)	8 (0.1%)	17.0%	38 (0.1%)	9 (0.1%)	19.1%
White	15066 (44.5%)	2319 (29.7%)	13.3%	15616 (46%)	1769 (23%)	10.2%
Multiple selected	1388 (4.1%)	152 (1.9%)	9.9%	1401 (4.1%)	139 (1.8%)	9.0%
My race is not listed	485 (1.4%)	58 (0.7%)	10.7%	482 (1.4%)	61 (0.8%)	11.2%
I prefer not to answer	1106 (3.3%)	965 (12.4%)	46.6%	978 (2.9%)	1093 (14.2%)	52.8%
Null response	469 (1.4%)	2083 (26.7%)	81.6%	428 (1.3%)	2124 (27.6%)	83.2%
Age tier at registration						
18–26	4335 (12.8%)	809 (10.4%)	15.7%	4242 (12.5%)	902 (11.7%)	17.5%
27–35	13211 (39%)	2366 (30.3%)	15.2%	13047 (38.4%)	2530 (32.8%)	16.2%
36–45	10107 (29.8%)	2482 (31.8%)	19.7%	10165 (29.9%)	2424 (31.5%)	19.3%
46–55	4554 (13.4%)	1375 (17.6%)	23.2%	4700 (13.8%)	1229 (16%)	20.7%
56–64	1478 (4.4%)	671 (8.6%)	31.2%	1598 (4.7%)	551 (7.2%)	25.6%
65+	194 (0.6%)	95 (1.2%)	32.9%	221 (0.7%)	68 (0.9%)	23.5%
Geographic region						
East North Central	2081 (6.1%)	687 (8.8%)	24.8%	2195 (6.5%)	573 (7.4%)	20.7%
East South Central	672 (2%)	253 (3.2%)	27.4%	696 (2%)	229 (3%)	24.8%
Middle Atlantic	4723 (13.9%)	804 (10.3%)	14.5%	4724 (13.9%)	803 (10.4%)	14.5%
Mountain	1458 (4.3%)	444 (5.7%)	23.3%	1522 (4.5%)	380 (4.9%)	20.0%
New England	1106 (3.3%)	249 (3.2%)	18.4%	1113 (3.3%)	242 (3.1%)	17.9%
Pacific	17290 (51%)	3009 (38.6%)	14.8%	16844 (49.6%)	3455 (44.8%)	17.0%
South Atlantic	3164 (9.3%)	1156 (14.8%)	26.8%	3347 (9.9%)	973 (12.6%)	22.5%
West North Central	784 (2.3%)	324 (4.2%)	29.2%	860 (2.5%)	248 (3.2%)	22.4%
West South Central	2601 (7.7%)	872 (11.2%)	25.1%	2672 (7.9%)	801 (10.4%)	23.1%

SOURCE Authors’ analysis of member responses to optional questions during digital health application registration.

^a^All differences between gender identity respondents and nonrespondents and sexual orientation respondents and nonrespondents were statistically significant at p<0.0001. Nonresponse rates = (I prefer not to answer responses + null responses)/all newly registered members who continued to optional questions.

Among members, sexual orientation nonresponse rates ranged from 16% (ages 27–35) to 26% (ages 56–64), while gender identity nonresponse rates ranged from 15% (ages 27–35) to 33% (ages 65+). Nonresponse rates for sexual orientation and gender identity were lowest in the Middle Atlantic (15%) and the Pacific (17% and 15%, respectively), but were higher in central and southern regions, ranging from 21–25% for sexual orientation and 25–29% for gender identity.

SOGI nonresponse rates differed across races and ethnicities. Members who selected multiple races and ethnicities had the lowest nonresponse rates for sexual orientation and gender identity at 9% and 10%, respectively. Among those who provided race and ethnicity information, American Indian or Alaska Native members had the highest nonresponse rates with 19% for sexual orientation and 27% for gender identity. For sexual orientation, Native Hawaiian or Other Pacific Islander members also had a high nonresponse rate of 19%.

Recognizing that motivations could vary between members who responded ’I prefer not to answer’ and members with null responses, we conducted a sensitivity test. This test excluded members who chose ’I prefer not to answer’ and considered only ’null responses’ as nonresponses. The results were consistent to those presented above; differences between respondents and nonrespondents remained statistically significant (S1 Table).

## Discussion

To our knowledge, this is the first study to examine response rates to optional SOGI questions within a digital health application. Our study sample represents a diverse group of over 41,000 commercially-insured adults who used a digital health application for virtual care and care navigation services. Adults were located in all 50 states with less than half identifying as White. We found that almost 82% of this diverse group of adults were willing to share their sexual orientation and gender identity during the registration process. These results contribute to the growing body of literature that adults are willing to share their sexual orientation and gender identity with health care entities, including through a digital health application.

We found that the proportion who chose ‘I prefer not to answer’, 7.9% for sexual orientation and 5.3% for gender identity, were within the range of other self-administered surveys. For example, one study found that 12% of patients at urology or cancer clinics refused to answer questions about their sexual orientation, and 7% refused to answer questions about their gender identity [12]. Another study of 293 patients at outpatient clinics had a 5% refusal rate for sexual orientation and 0.3% for gender identity [11]. While our findings are consistent with these studies in terms of nonresponse rates, the magnitude of nonresponse was closer between the two questions in our study. Drivers of this difference are unknown, but two possible explanations may be differences in study samples and study time periods. For example, our study sample was much larger and more diverse in terms of demographic characteristics. Additionally, societal attitudes towards sexual orientation and gender identity have changed over time, which may have contributed to similar rates of nonresponse in our study compared to earlier studies.

Our study found that SOGI nonresponse rates varied by demographic characteristics, similar to previous research. We discovered that gender identity refusal rates increased with age, aligning with Rullo et al.’s findings [11]. Moreover, we observed higher nonresponse rates among individuals residing in states where legislators are approving or introducing anti-LGBTQ+ bills [24]. This finding is consistent with a prior study, which found that SOGI data collection was higher in cities with protective SOGI nondiscrimination policies [18]. The reasons behind the higher nonresponse rates among people residing in areas of the country where state legislators are introducing or approving anti-LGBTQ+ bills are unclear. It may be due to fear of self-identification among members of SGM communities or antagonism towards the SGM community. In our data, the proportion of respondents who identified as a member of a SGM community was similar across geographies, indicating fear of self-identification may not differ by geography. However, further research is necessary to fully understand the underlying factors.

Our study also identified that nonresponse rates varied by race and ethnicity, with the highest nonresponse rates among Black, Native Hawaiian or Other Pacific Islander, and American Indian or Alaska Native adults. White adults had lower nonresponse rates for sexual orientation, but higher nonresponse rates for gender identity compared to Asian adults and those whose race was not listed. Although no studies have compared SOGI nonresponse rates by race and ethnicity from self-administered surveys, unadjusted results from two studies based on the SOGI module of the Behavioral Risk Factor Surveillance System (BRFSS) survey produced mixed findings. One study, based on seven states, reported that non-Hispanic White adults had the lowest gender identity nonresponse rate, but adults of multiple races or non-Hispanic other race had lower nonresponse rates than White adults for sexual orientation [19]. The other study, based only on Washington BRFSS, found that Hispanic and Asian adults had the highest sexual orientation nonresponse rates, whereas non-Hispanic White adults had the lowest [20].

This study is not without limitations. First, results lack generalizability beyond commercially-insured adults who used a digital health application provided through their employer. Additionally, the response rates and distributions may differ if these questions were asked of all members who registered instead of only those who opted to continue to additional questions. The study is also limited by the lack of complete demographic data on the eligible population, which limits our understanding of differences between respondents and nonrespondents. Another limitation is that while the SOGI questions used in the study were informed by validated instruments, the two-step gender identity question was not used. Instead, we asked about current gender identity to reduce member friction, which may have increased comprehension issues with the response options presented. However, we included definitions of each response option within the application to increase member understanding. Finally, the expansive and ever-changing nature of SOGI categories may have increased nonresponse among members whose identity is not included. However, the option to select ‘my identity/gender is not listed’ likely minimized this limitation.

To increase the collection of SOGI data in digital applications, survey structure is a crucial consideration. We identified three potential changes for future consideration and research. First, the survey should be presented to as many members as possible to reduce the drop in responsiveness due to flow design. We found that 63% of potential respondents were lost before they even saw the questions. Second, including a ‘don’t know/not sure’ field provides a response option for members who may not understand the options presented. Finally, to counteract concerns around confidentiality and data use highlighted in the literature, the digital application could prominently display the purpose and secure storage of the data being collected [12,25].

## Conclusion

This study highlights the potential of digital health applications to collect SOGI data from a diverse group of commercially-insured adults. Further research is needed to identify strategies to increase SOGI response rates and understand the underlying drivers of nonresponse. As digital health applications become more prevalent, they can be leveraged as a tool to collect critical SOGI data for delivering equitable care and enabling future research and improvement initiatives.

## Supporting information

S1 Table Characteristics of members by gender identity and sexual orientation response, between September 9th and December 31, 2022. (DOCX)

S1 File STROBE checklist. (DOCX)

S2 File Study minimal dataset. (CSV)

## References

[1] Zeeman L, Sherriff N, Browne K, McGlynn N, Mirandola M, Gios L, et al. A review of lesbian, gay, bisexual, trans and intersex (LGBTI) health and healthcare inequalities. European Journal of Public Health. 2019 Oct 1;29(5):974–80. doi: 10.1093/eurpub/cky226 30380045 PMC6761838

[2] Institute of Medicine, Committee on Lesbian, Gay, Bisexual, and Transgender Health Issues and Research Gaps and Opportunities, Garofalo R. The health of lesbian, gay, bisexual, and transgender people: Building a foundation for better understanding. Washington, DC: The National Academies Press, 2011. 347 p. doi: 10.17226/13128 22013611

[3] Liu M, Sandhu S, Keuroghlian AS. Achieving the triple aim for sexual and gender minorities. New England Journal of Medicine. 2022 Jul 28;387(4):294–7. doi: 10.1056/NEJMp2204569 35866741

[4] Cahill SR. Still in the dark regarding the public health impact of COVID-19 on sexual and gender minorities. American Journal of Public Health. 2021 Sep;111(9):1606–9. doi: 10.2105/AJPH.2021.306397 34410865 PMC8589066

[5] Joint Commission. Advancing effective communication, cultural competence, and patient- and family-centered care for the lesbian, gay, bisexual, and transgender community: A field guide. Oakbrook Terrace, IL: Joint Commission; 2011. Available from: https://www.jointcommission.org/-/media/tjc/documents/resources/patient-safety-topics/health-equity/lgbtfieldguide_web_linked_verpdf.pdf?db=web&hash=FD725DC02CFE6E4F21A35EBD839BBE97.

[6] Cahill SR, Baker K, Deutsch MB, Keatley J, Makadon HJ. Inclusion of sexual orientation and gender identity in stage 3 meaningful use guidelines: a huge step forward for LGBT health. LGBT Health. 2016 Apr 1;3(2):100–2. doi: 10.1089/lgbt.2015.0136 26698386

[7] National Academies of Sciences, Engineering, and Medicine. Measuring sex, gender identity, and sexual orientation. 2022 Mar 9. 10.17226/26424. 35286054

[8] Broekhuis JM, Cloonan DJ. A Call for the collection of data on sexual orientation and gender identity for surgical research—What we don’t know can hurt us. JAMA Surgery. 2023 Feb 1;158(2):111–2. doi: 10.1001/jamasurg.2022.3950 36260327

[9] MacCarthy S, Elliott MN. Sexual orientation And gender identity data. Health Affairs. 2021 May 1;40(5):852. doi: 10.1377/hlthaff.2021.00255 33939514

[10] Cahill S, Singal R, Grasso C, King D, Mayer K, Baker K, et al. Do ask, do tell: high levels of acceptability by patients of routine collection of sexual orientation and gender identity data in four diverse American community health centers. PLOS ONE. 2014 Sep 8;9(9):e107104. doi: 10.1371/journal.pone.0107104 25198577 PMC4157837

[11] Rullo JE, Foxen JL, Griffin JM, Geske JR, Gonzalez CA, Faubion SS, van Ryn M. Patient acceptance of sexual orientation and gender identity questions on intake forms in outpatient clinics: a pragmatic randomized multisite trial. Health Services Research. 2018 Oct;53(5):3790–808. doi: 10.1111/1475-6773.12843 29522236 PMC6153164

[12] Rosser BS, Polter EJ, Chandiramani N, Cahill S, Wheldon CW, Konety BR, et al. Acceptability and feasibility of collecting sexual orientation and expanded gender identity data in urology and oncology clinics. LGBT Health. 2021 Sep 1;8(6):420–6. doi: 10.1089/lgbt.2020.0256 34348045 PMC8591059

[13] Haider AH, Schneider EB, Kodadek LM, Adler RR, Ranjit A, Torain M, et al. Emergency department query for patient-centered approaches to sexual orientation and gender identity: the EQUALITY study. JAMA Internal Medicine. 2017 Jun 1;177(6):819–28. doi: 10.1001/jamainternmed.2017.0906 28437523 PMC5818827

[14] Ruben MA, Blosnich JR, Dichter ME, Luscri L, Shipherd JC. Will veterans answer sexual orientation and gender identity questions? Medical Care. 2017 Sep 1;55(9):S85–9. doi: 10.1097/MLR.0000000000000744 28806370

[15] U.S. Food and Drug Administration. What is digital health? September 22, 2020. Available from: https://www.fda.gov/medical-devices/digital-health-center-excellence/what-digital-health.

[16] Eastburn J, Harris A, Nagarajan N, Rost J. Is virtual care delivering on it’s promise of improving access?. McKinsey and Associates; Jan 9 2023. Available from: https://www.mckinsey.com/industries/healthcare/our-insights/is-virtual-care-delivering-on-its-promise-of-improving-access.

[17] Globe Newswire. Global digital health market report 2020: Market is expected to witness a 37.1% spike in growth in 2021 and will continue to grow and reach USD 508.8 billion by 2027. November 25, 2020. Available from: https://www.globenewswire.com/news-release/2020/11/25/2133473/0/en/Global-Digital-Health-Market-Report-2020-Market-is-Expected-to-Witness-a-37-1-Spike-in-Growth-in-2021-and-will-Continue-to-Grow-and-Reach-US-508-8-Billion-by-2027.html.

[18] Almazan AN, King D, Grasso C, Cahill S, Lattanner M, Hatzenbuehler ML, et al. Sexual orientation and gender identity data collection at US health centers: impact of city-level structural stigma in 2018. American Journal of Public Health. 2021 Nov;111(11):2059–63. doi: 10.2105/AJPH.2021.306414 34499534 PMC8630472

[19] O’Brien RP, Blosnich JR. Refusal rates to sexual orientation and gender identity items in the behavioral risk factor surveillance system, 2014–2019. American Journal of Public Health. 2022 Mar;112(3):443–52. doi: 10.2105/AJPH.2021.306625 35196048 PMC8887176

[20] Kim HJ, Fredriksen-Goldsen KI. Nonresponse to a question on self-identified sexual orientation in a public health survey and its relationship to race and ethnicity. American Journal of Public Health. 2013 Jan;103(1):67–9. doi: 10.2105/AJPH.2012.300835 23153153 PMC3518335

[21] Vanderbilt University. How to ask about sexuality/gender. Available from: https://www.vanderbilt.edu/lgbtqi/resources/how-to-ask-about-sexuality-gender.

[22] Suen LW, Lunn MR, Katuzny K, Finn S, Duncan L, Sevelius J, et al. What sexual and gender minority people want researchers to know about sexual orientation and gender identity questions: A qualitative study. Archives of Sexual Behavior. 2020 Oct;49:2301–18. doi: 10.1007/s10508-020-01810-y 32875381 PMC7497435

[23] National Oceanic and Atmospheric Administration, National Centers for Environmental Information. U.S. Census Divisions. Available from: https://www.ncei.noaa.gov/access/monitoring/reference-maps/us-census-divisions.

[24] ACLU. Mapping attacks on LGBTQ rights in U.S. state legislatures [internet]. Available from: https://www.aclu.org/legislative-attacks-on-lgbtq-rights.

[25] Ogden SN, Scheffey KL, Blosnich JR, Dichter ME. “Do I feel safe revealing this information to you?”: Patient perspectives on disclosing sexual orientation and gender identity in healthcare. Journal of American College Health. 2020 Aug 17;68(6):617–23. doi: 10.1080/07448481.2019.1583663 32897171

