# Supplementary material for: Adults’ willingness to report sexual orientation and gender identity when registering for a digital health application: A cross-sectional quantitative study
Source: PLoS One. 2023 Nov 20;18(11):e0292739. doi: 10.1371/journal.pone.0292739 (PMC10659155; doi:10.1371/journal.pone.0292739)
Supplement: S1 Table — (DOCX) [file pone.0292739.s001.docx]

### **S2 Table. Characteristics of Members by Gender Identity and Sexual Orientation Response, between September 9th and December 31, 2022.**

|  | Gender identity | | | Sexual orientation | | |
| --- | --- | --- | --- | --- | --- | --- |
|  | Responded with gender identity (N) | Null response (N) | I prefer not to answer (N) | Responded with sexual orientation (N) | Null response (N) | I prefer not to answer (N) |
| Total | 33879 (100%) | 5582 (100%) | 2216 (100%) | 33973 (100%) | 4427 (100%) | 3277 (100%) |
| Race/ethnicity |  |  |  |  |  |  |
| American Indian or Alaska Native | 90 (0.3%) | 21 (0.4%) | 13 (0.6%) | 100 (0.3%) | 12 (0.3%) | 12 (0.4%) |
| Asian | 10786 (31.8%) | 748 (13.4%) | 534 (24.1%) | 10303 (30.3%) | 745 (16.8%) | 1020 (31.1%) |
| Black or African American | 2072 (6.1%) | 311 (5.6%) | 132 (6%) | 2139 (6.3%) | 193 (4.4%) | 183 (5.6%) |
| Hispanic | 2378 (7%) | 288 (5.2%) | 166 (7.5%) | 2488 (7.3%) | 145 (3.3%) | 199 (6.1%) |
| Native Hawaiian or Other Pacific Islander | 39 (0.1%) | 6 (0.1%) | 2 (0.1%) | 38 (0.1%) | 3 (0.1%) | 6 (0.2%) |
| White | 15066 (44.5%) | 1627 (29.1%) | 692 (31.2%) | 15616 (46%) | 787 (17.8%) | 982 (30%) |
| Multiple selected | 1388 (4.1%) | 104 (1.9%) | 48 (2.2%) | 1401 (4.1%) | 56 (1.3%) | 83 (2.5%) |
| My race is not listed | 485 (1.4%) | 33 (0.6%) | 25 (1.1%) | 482 (1.4%) | 25 (0.6%) | 36 (1.1%) |
| I prefer not to answer | 1106 (3.3%) | 380 (6.8%) | 585 (26.4%) | 978 (2.9%) | 362 (8.2%) | 731 (22.3%) |
| Null response | 469 (1.4%) | 2064 (37%) | 19 (0.9%) | 428 (1.3%) | 2099 (47.4%) | 25 (0.8%) |
| Age tier at registration, y |  |  |  |  |  |  |
| 18-26 | 4335 (12.8%) | 614 (11%) | 195 (8.8%) | 4242 (12.5%) | 530 (12%) | 372 (11.4%) |
| 27-35 | 13211 (39%) | 1758 (31.5%) | 608 (27.4%) | 13047 (38.4%) | 1497 (33.8%) | 1033 (31.5%) |
| 36-45 | 10107 (29.8%) | 1762 (31.6%) | 720 (32.5%) | 10165 (29.9%) | 1401 (31.6%) | 1023 (31.2%) |
| 46-55 | 4554 (13.4%) | 913 (16.4%) | 462 (20.8%) | 4700 (13.8%) | 640 (14.5%) | 589 (18%) |
| 56-64 | 1478 (4.4%) | 475 (8.5%) | 196 (8.8%) | 1598 (4.7%) | 324 (7.3%) | 227 (6.9%) |
| 65+ | 194 (0.6%) | 60 (1.1%) | 35 (1.6%) | 221 (0.7%) | 35 (0.8%) | 33 (1%) |
| Geographic region |  |  |  |  |  |  |
| East North Central | 2081 (6.1%) | 517 (9.3%) | 170 (7.7%) | 2195 (6.5%) | 350 (7.9%) | 223 (6.8%) |
| East South Central | 672 (2%) | 184 (3.3%) | 69 (3.1%) | 696 (2%) | 128 (2.9%) | 101 (3.1%) |
| Middle Atlantic | 4723 (13.9%) | 569 (10.2%) | 235 (10.6%) | 4724 (13.9%) | 480 (10.8%) | 323 (9.9%) |
| Mountain | 1458 (4.3%) | 329 (5.9%) | 115 (5.2%) | 1522 (4.5%) | 234 (5.3%) | 146 (4.5%) |
| New England | 1106 (3.3%) | 185 (3.3%) | 64 (2.9%) | 1113 (3.3%) | 143 (3.2%) | 99 (3%) |
| Pacific | 17290 (51%) | 2063 (37%) | 946 (42.7%) | 16844 (49.6%) | 1881 (42.5%) | 1574 (48%) |
| South Atlantic | 3164 (9.3%) | 867 (15.5%) | 289 (13%) | 3347 (9.9%) | 602 (13.6%) | 371 (11.3%) |
| West North Central | 784 (2.3%) | 234 (4.2%) | 90 (4.1%) | 860 (2.5%) | 149 (3.4%) | 99 (3%) |
| West South Central | 2601 (7.7%) | 634 (11.4%) | 238 (10.7%) | 2672 (7.9%) | 460 (10.4%) | 341 (10.4%) |

### 
