# Supplementary material for: Adults’ willingness to report sexual orientation and gender identity when registering for a digital health application: A cross-sectional quantitative study
Source: PLoS One. 2023 Nov 20;18(11):e0292739. doi: 10.1371/journal.pone.0292739 (PMC10659155; doi:10.1371/journal.pone.0292739)
Supplement: S1 File — (DOCX) [file pone.0292739.s002.docx]

## S1 File. STROBE Statement.

|  | **Item No.** | **Recommendation** | **Page  No.** | **Relevant text from manuscript** |
| --- | --- | --- | --- | --- |
| **Title and abstract** | 1 | (*a*) Indicate the study’s design with a commonly used term in the title or the abstract | 1 | Adults’ willingness to report sexual orientation and gender identity when registering or a digital health application: A cross-sectional quantitative study |
|  |  | (*b*) Provide in the abstract an informative and balanced summary of what was done and what was found | 2 | We analyzed response rates for sexual orientation and gender identity by age, race and ethnicity, and region among individuals aged 18 years or older between September 9th and December 31, 2022. Our study, which included over 41,000 commercially-insured adults from all 50 states, found that nearly 80% were willing to report their sexual orientation and gender identity. However, we observed higher nonresponse rates among older adults and individuals living in central and southern regions, with no consistent patterns by race and ethnicity. |
| **Introduction** | | | |  |
| Background/rationale | 2 | Explain the scientific background and rationale for the investigation being reported | 4 | While research has shown that patients are willing to share this information, especially in outpatient settings, it is unknown if they would be willing to report SOGI data through digital health applications. |
| Objectives | 3 | State specific objectives, including any prespecified hypotheses | 4 | The purpose of this study was to determine the likelihood of adults to provide their sexual orientation and gender identity during the enrollment process of a digital health application. It also sought to test our hypotheses that SOGI response rates are lower among members in older age groups, members who reside in the southern and central regions of the US, and were not White. |
| **Methods** | | | |  |
| Study design | 4 | Present key elements of study design early in the paper | 5 | See next rows |
| Setting | 5 | Describe the setting, locations, and relevant dates, including periods of recruitment, exposure, follow-up, and data collection | 5 | The digital health application includes both health care navigation and virtual care services offered as an employer-provided benefit to employees and their dependents (‘members’).  Members may register for the application at any point after the employer begins to cover the benefit. As of September 9th, 2022, we began collecting optional demographic information during the registration process. Our study sample included all members over 17 years old who registered in the application on or after September 9th through December 31, 2022. |
| Participants | 6 | (*a*) *Cohort study*—Give the eligibility criteria, and the sources and methods of selection of participants. Describe methods of follow-up  *Case-control study*—Give the eligibility criteria, and the sources and methods of case ascertainment and control selection. Give the rationale for the choice of cases and controls  *Cross-sectional study*—Give the eligibility criteria, and the sources and methods of selection of participants | 5 | See prior row |
|  |  | (*b*) *Cohort study*—For matched studies, give matching criteria and number of exposed and unexposed  *Case-control study*—For matched studies, give matching criteria and the number of controls per case | n/a |  |
| Variables | 7 | Clearly define all outcomes, exposures, predictors, potential confounders, and effect modifiers. Give diagnostic criteria, if applicable | 6-8 | The SOGI questions and response options were informed by validated instruments and expanded for greater inclusivity (SOGI questions and response options included in text). For race and ethnicity, sexual orientation, and pronouns, members were allowed to choose multiple response options. In these cases, we categorized the response as 'multiple select' and did not include the responses within the categories they selected. We made this analytical decision to ensure mutually exclusive groups for statistical testing. In addition, members who chose ‘my race/pronouns/gender/identity is not listed’ and then entered free text that was similar to a predefined option, were not recategorized. The responses remained coded as ‘my race/pronouns/gender/identity is not listed’. Members who chose to not answer the question (missing values) were coded as ‘null response’.  We defined item nonresponse as anyone who responded ‘I prefer not to answer’ or had a ‘null response’. |
| Data sources/ measurement | 8* | For each variable of interest, give sources of data and details of methods of assessment (measurement). Describe comparability of assessment methods if there is more than one group | 7 | The variables included member responses to the four optional questions above and member age and geographic location from the employer eligibility files. |
| Bias | 9 | Describe any efforts to address potential sources of bias |  |  |
| Study size | 10 | Explain how the study size was arrived at | 5 | Our study sample included all members over 17 years old who registered in the application on or after between September 9th through December 31, 2022. |

Continued on next page

|  | **Item No.** | **Recommendation** | **Page  No.** | **Relevant text from manuscript** |  |
| --- | --- | --- | --- | --- | --- |
| Quantitative variables | 11 | Explain how quantitative variables were handled in the analyses. If applicable, describe which groupings were chosen and why | 8 | Age was defined as 18 to 26, 27 to 35, 36 to 45, 46 to 55, 56 to 64, or 65+. |  |
| Statistical methods | 12 | (*a*) Describe all statistical methods, including those used to control for confounding | 8 | We calculated chi-square statistics to test for correlation in item nonresponse (‘I prefer not to answer’ or ‘null response’) across race and ethnicity, gender identity, and sexual orientation questions. We also used chi-square statistics to test for differences in race and ethnicity, age, and geographic location between members who responded to individual SOGI questions and members who did not. We calculated nonresponse rates overall and separately by race and ethnicity, age, and geographic location. |  |
|  |  | (*b*) Describe any methods used to examine subgroups and interactions | n/a |  |  |
|  |  | (*c*) Explain how missing data were addressed | 8 | Members who chose to not answer the question (missing values) were coded as ‘null response’. |  |
|  |  | (*d*) *Cohort study*—If applicable, explain how loss to follow-up was addressed  *Case-control study*—If applicable, explain how matching of cases and controls was addressed  *Cross-sectional study*—If applicable, describe analytical methods taking account of sampling strategy | n/a |  |  |
|  |  | (*e*) Describe any sensitivity analyses | 8-9 | We also conducted a sensitivity test that removed ‘I prefer not to answer’ responses from the nonresponse group and recalculated the chi-square statistic between respondents and nonrespondents to see if any differences found in the primary comparison remained. |  |
| **Results** | | | | |  |
| Participants | 13* | (a) Report numbers of individuals at each stage of study—eg numbers potentially eligible, examined for eligibility, confirmed eligible, included in the study, completing follow-up, and analysed | 9 | Overall, 113,064 new members registered during the study period and 41,677 (36.8%) chose to continue to the optional questions |  |
|  |  | (b) Give reasons for non-participation at each stage | n/a |  |  |
|  |  | (c) Consider use of a flow diagram | n/a |  |  |
| Descriptive data | 14* | (a) Give characteristics of study participants (eg demographic, clinical, social) and information on exposures and potential confounders | 9-12 | Table 2 includes the responses to the questions and the member demographic characteristics among those presented with the optional questions. |  |
|  |  | (b) Indicate number of participants with missing data for each variable of interest | 11-12 | The race and ethnicity nonresponse rate was 11%; 5% chose ‘I prefer not to answer’ and 6% had a null response. Fifty-one percent of members selected he/him/his pronouns, 39% chose she/her/hers, and 1.1% selected multiple responses. The nonresponse rate was 7.7%; 0.25% chose ‘I prefer not to answer’ and 7% had a null response.Over 79% of members’ gender identity was cisgender, 1.2% of members’ gender was not listed, and 1.4% identified as transgender, agender, genderfluid or non-binary. The nonresponse rate was 18.7%; A total of 18.7% did not respond; 5.3% chose ‘I prefer not to answer’ and 13.4% had a null response.  Over 18% did not provide their sexual orientation; 7.9% chose ‘I prefer not to answer’ and 10.6% had a null response. |  |
|  |  | (c) *Cohort study*—Summarise follow-up time (eg, average and total amount) | n.a |  |  |
| Outcome data | 15* | *Cohort study*—Report numbers of outcome events or summary measures over time | n/a |  |  |
|  |  | *Case-control study—*Report numbers in each exposure category, or summary measures of exposure | n/a |  |  |
|  |  | *Cross-sectional study—*Report numbers of outcome events or summary measures | 9-12 | Table 2 includes the responses to the questions and the member demographic characteristics among those presented with the optional questions. |  |
| Main results | 16 | (*a*) Give unadjusted estimates and, if applicable, confounder-adjusted estimates and their precision (eg, 95% confidence interval). Make clear which confounders were adjusted for and why they were included | 12-14 | Table 3 displays nonresponse rates by member demographics and compares characteristics of sexual orientation and gender identity respondents to nonrespondents. Respondents who provided information about their sexual orientation and gender identity exhibited differences from nonrespondents in terms of race and ethnicity, age, and geographic region (p<0.001). SOGI nonresponse rates were higher among older members, members residing in the central and southern U.S regions, and varied across different races and ethnicities |  |
|  |  | (*b*) Report category boundaries when continuous variables were categorized | 9-11 | Age was reported with category boundaries in Table 2. |  |
|  |  | (*c*) If relevant, consider translating estimates of relative risk into absolute risk for a meaningful time period | n/a |  |  |

Continued on next page

| Other analyses | 17 | Report other analyses done—eg analyses of subgroups and interactions, and sensitivity analyses | 15 | The association between nonresponse rates to race and ethnicity, sexual orientation, and gender identity questions was statistically significant (p<0.0001). Recognizing that motivations could vary between members who responded 'I prefer not to answer' and members with null responses, we conducted a sensitivity test. This test excluded members who chose 'I prefer not to answer' and considered only 'null responses' as nonresponses. The results were consistent to those presented above; differences between respondents and nonrespondents remained statistically significant along with the characteristics associated with a higher likelihood of nonresponse (S2 Table). |
| --- | --- | --- | --- | --- |
| **Discussion** | | | | |
| Key results | 18 | Summarise key results with reference to study objectives | 15 | Our study sample represents a diverse group of over 41,000 commercially-insured adults who used a digital health application for virtual care and care navigation services. Adults were located in all 50 states with less than half identifying as White. We found that almost 82% of this diverse group of adults were willing to share their sexual orientation and gender identity during the registration process. These results contribute to the growing body of literature that adults are willing to share their sexual orientation and gender identity with health care entities, including through a digital health application. |
| Limitations | 19 | Discuss limitations of the study, taking into account sources of potential bias or imprecision. Discuss both direction and magnitude of any potential bias | 17 | First, results lack generalizability beyond commercially-insured adults who used a digital health application provided through their employer. Additionally, the response rates and distributions may differ if these questions were asked of all members who registered instead of only those who opted to continue to additional questions. The study is also limited by the lack of complete demographic data on the eligible population, which limits our understanding of differences between respondents and nonrespondents. Another limitation is that while the SOGI questions used in the study were informed by validated instruments, the two-step gender identity question was not used. Instead, we asked about current gender identity to reduce member friction, which may have increased comprehension issues with the response options presented. However, we included definitions of each response option within the application to increase member understanding. Finally, the expansive and ever-changing nature of SOGI categories may have increased nonresponse among members whose identity is not included. However, the option to select ‘my identity/gender is not listed’ likely minimized this limitation. |
| Interpretation | 20 | Give a cautious overall interpretation of results considering objectives, limitations, multiplicity of analyses, results from similar studies, and other relevant evidence | 15-17 | See Discussion section. |
| Generalisability | 21 | Discuss the generalisability (external validity) of the study results | 17 | results lack generalizability beyond commercially-insured adults who used a digital health application provided through their employer. |
| **Other information** | |  | | |
| Funding | 22 | Give the source of funding and the role of the funders for the present study and, if applicable, for the original study on which the present article is based |  | See funding source and statement submitted with manuscript. |

*Give information separately for cases and controls in case-control studies and, if applicable, for exposed and unexposed groups in cohort and cross-sectional studies.

**Note:** An Explanation and Elaboration article discusses each checklist item and gives methodological background and published examples of transparent reporting. The STROBE checklist is best used in conjunction with this article (freely available on the Web sites of PLoS Medicine at http://www.plosmedicine.org/, Annals of Internal Medicine at http://www.annals.org/, and Epidemiology at http://www.epidem.com/). Information on the STROBE Initiative is available at www.strobe-statement.org.
